# 

**Published:** 1854-02

**Authors:** 


A Monograph on the Functional and Sympathetic Affections of the Heart, by John M. Corson, M. D., of New York, has been issued in pamphlet form. This paper has previously appeared in the New York Journal of Medicine. It is carefully written, and from the very cursory examination we have been able to give it, it seems a very faithful *resume* of the present state of information on that subject. H.

---

The American Medical Monthly is a new periodical just started in New York, published by Putnam, well furnished with capital, conducted by the Faculty of the New York Medical College, and under the special editorial supervision of Edward H. Parker, M. D. With such resources it can scarcely fail of success. H.

Our readers will notice, that our editorial department is this month devoted to the disposition of a portion of the flood of pamphlets, and introductories, which have reached us. Most of these it was an easy matter to read, and a pleasure to commend. Our annual introductory lectures before college classes are, in some measure, an index of the prevailing medical sentiment. This gives them value as a record of the times, and makes them—aside from their merits as literary compositions—worthy of the critics notice.

